# a-SiN_x_:H-based ultra-low power resistive random access memory with tunable Si dangling bond conduction paths

**DOI:** 10.1038/srep15762

**Published:** 2015-10-28

**Authors:** Xiaofan Jiang, Zhongyuan Ma, Jun Xu, Kunji Chen, Ling Xu, Wei Li, Xinfan Huang, Duan Feng

**Affiliations:** 1 School of Electronic Science and Engineering, Nanjing University, Nanjing, 210093, China; 2 Collaborative Innovation Center of Advanced Microstructures, Nanjing University, Nanjing, 210093, China; 3 Jiangsu Provincial Key Laboratory of Photonic and Electronic Materials Sciences and Technology, Nanjing University, Nanjing, 210093, China

## Abstract

The realization of ultra-low power Si-based resistive switching memory technology will be a milestone in the development of next generation non-volatile memory. Here we show that a high performance and ultra-low power resistive random access memory (RRAM) based on an Al/a-SiN_x_:H/p^+^-Si structure can be achieved by tuning the Si dangling bond conduction paths. We reveal the intrinsic relationship between the Si dangling bonds and the N/Si ratio x for the a-SiN_x_:H films, which ensures that the programming current can be reduced to less than 1 μA by increasing the value of x. Theoretically calculated current-voltage (*I–V *) curves combined with the temperature dependence of the *I–V* characteristics confirm that, for the low-resistance state (LRS), the Si dangling bond conduction paths obey the trap-assisted tunneling model. In the high-resistance state (HRS), conduction is dominated by either hopping or Poole–Frenkel (P–F) processes. Our introduction of hydrogen in the a-SiN_x_:H layer provides a new way to control the Si dangling bond conduction paths, and thus opens up a research field for ultra-low power Si-based RRAM.

As device sizes continue to be scaled down, conventional flash memory is expected to reach its physical size limits in the near future. As a potential replacement for flash memory, resistive random access memory (RRAM) has attracted considerable attention because of advantages that include a simple fabrication process, fast operating speeds, long retention times and good scalability^1^,^2^,^3^,^4^. However, the normal programming currents of RRAMs (>10 μA) are still much higher than those required for flash memory (<10 nA)^5^,^6^, and thus reduction of the device current and power consumption is an important issue for RRAM applications. The lowest reported reset current for RRAM to date is approximately 1 μA when using the high-k material Al_2_O_3_^7^, and this result was ascribed to the low defect density of the Al_2_O_3_ film. In contrast to resistive switching memories based on high-k materials, resistive switching (RS) in low power Si-based devices is a more attractive and promising prospect because of the full compatibility of these devices with traditional complementary metal-oxide-semiconductor (CMOS) technology. Among the available Si-based RRAM materials, amorphous-SiN_x_ (a-SiN_x_) films exhibit more stable RS behavior at lower operating voltages than conventional SiO_x_ films^8^,^9^,^10^,^11^,^12^,^13^,^14^. It should be noted that the programming currents of a-SiN_x_-based devices are higher (~100 μA) because high numbers of random traps make the pristine a-SiN_x_ films leakier. Finding an effective way to control the conduction paths in these films has been a challenging issue. To reduce the power of a-SiN_x_-based devices, we introduced hydrogen into the a-SiN_x_ film, which then shows greatly improved insulating properties with low leakage current because of the hydrogen passivation effect^15^. We found that the field-enhanced thermal breakage of weak Si-H bonds and the H^+^ ions migration result in the generation and re-passivation of Si dangling bonds in the a-SiN_x_:H films. Here we show a tunable dangling bond path effect on the programming current of a-SiN_x_:H based on variation of the N/Si ratio. By tuning the N/Si ratio x from 0.62 to 1.17, the number of dangling bonds can be reduced to produce programming currents of less than 1 μA. In addition, the electroforming and set operations of a-SiN_x_:H layers are easier than that required for Al_2_O_3_ and SiO_x_ layers because of the weak Si-H bond energy and the comparatively low dielectric breakdown strength of a-SiN_x_:H^16^. We demonstrate that an appropriate N/Si ratio can be used to control the concentrations of Si-H bonds and Si dangling bonds, which is the crucial factor in production of ultra-low power devices. Lower N/Si ratios result in increased numbers of Si dangling bonds and high programming currents, while high N/Si ratios will make the film too insulated and make the forming process difficult to be completed. The a-SiN_x_:H-based RRAM with optimum N/Si ratio is expected to be a suitable candidate for ultra-low power Si-based RRAMs.

## Results

### Programming current reduction by N/Si ratio tuning

**Figure 1:**
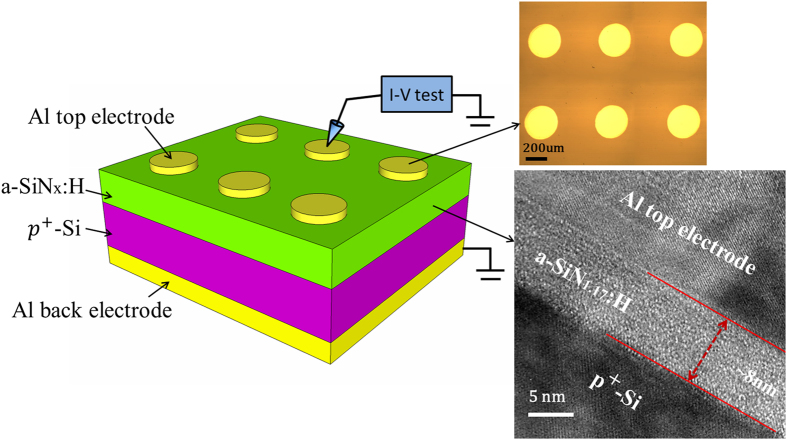
Schematic illustration of the a-SiN_x_:H-based RRAM device with electrical measurement. On the right, a photograph of the top electrodes and cross-sectional HRTEM image of the Al/a-SiN_1.17_:H/p^+^-Si structure are also shown.

**Figure 2:**
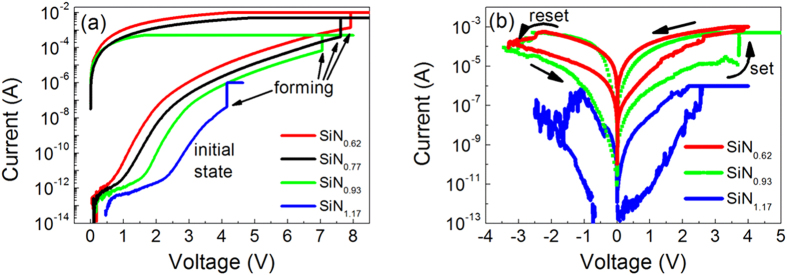
Effects of different N/Si ratios on RS behavior. (**a**) Initial current and forming curves of devices with N/Si ratios of 1.17, 0.93, 0.77 and 0.62. The initial current increases obviously with decreasing N/Si ratio. (**b**) *I-V* curves of RS behaviors after forming process. All devices show bipolar RS behavior and the current decrease substantially with increasing N/Si ratio.

A schematic of the Al/a-SiN_x_:H/p^+^-Si device structure is shown in Fig. 1. An approximately 8-nm-thick a-SiN_x_:H film was deposited on a p^+^-Si substrate. The substrate resistivity was in the 0.004–0.0075 Ω∙cm range, which ensured good conductivity as the bottom electrode. For the electrical measurements, we deposited aluminum (Al) as top and back electrode layers. A cross-sectional high-resolution transmission electron microscopy (HRTEM) image of the Al/a-SiN_1.17_:H/p^+^-Si structure is shown on the right of Fig. 1. During all the electrical measurements, the bias signal was applied to the top electrode with the back electrode grounded. Figure 2a shows the initial *I-V* curves and forming processes of four devices with different N/Si ratios. The ratio x is determined to be 1.17, 0.93, 0.77 and 0.62 for these devices through X-ray photoelectron spectroscopy (XPS) measurements. We notice that for all devices, the forming current value decreases by several orders with increasing x from 0.62 to 1.17. As indicated by Tao *et al*^17^, the bandgap energy of the a-SiN_x_:H film decreases with increasing Si concentration. The narrower bandgap causes more electrons or holes to be thermally excited to localized states near the conduction or valence bands because the carrier density is proportional to 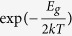, where *E*_*g*_ denotes the bandgap energy. Therefore, more charge carriers take part in conduction, which leads to increased initial currents. After the forming process, the devices with x = 0.62, 0.93 and 1.17 present bipolar RS behavior, as shown in Fig. 2b. The set and reset currents also decrease obviously with increasing x. For devices with x = 0.62–0.93, the programming current can be reduced from 1 mA to 10 μA. This value is close to that previously reported for SiN_x_-based RRAM^9^,^10^. The current also decreases further with increasing x, reaching a very low value of less than 1 μA when x = 1.17. This shows that we can control the current and power of a-SiN_x_:H-based RRAM devices by tuning the N/Si ratio.

### Bipolar switching performance of ultra-low power a-SiN_1.17_:H device

**Figure 3:**
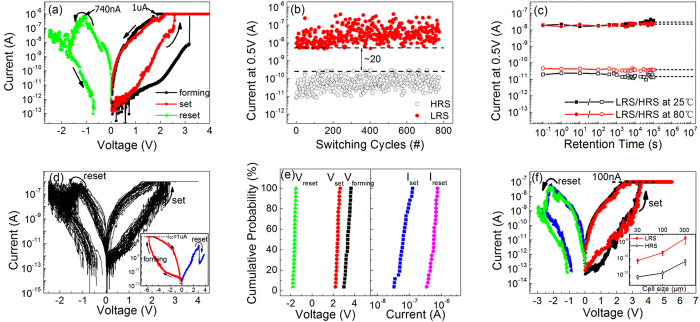
RS characteristics of the ultra-low power a-SiN_1.17_:H device. (**a**) Typical *I-V* curves of the bipolar RS behavior. Note that the set and reset currents are both less than 1 μA. (**b**) The endurance test of about 800 cycles using DC sweep. The minimum on/off ratio is approximately 20. (**c**) Retention characteristics of the device at room temperature and at 80 °C. (**d**) *I-V* curves containing 30 RS cycles. The inset shows measured *I-V* characteristics from negative forming. (**e**) Statistical result of operating voltages and current of 25 devices. (**f**) RS behavior when electrode size decreased to 30 μm. The inset shows size-dependence of LRS and HRS current from 300 μm to 30 μm. The error bar at each point is calculated by the standard deviation of 10 measurements.

The details of the memory performance of the SiN_1.17_:H device with the lowest measured current are shown in Fig. 3a. Polarity-dependent bipolar switching occurs for a compliance current of 1 μA when the bias voltage increases to 3.2 V. After the forming process, the current reaches a higher value and the device switches from the initial resistance state (IRS) to the low resistance state (LRS). A reset process can be achieved when a negative bias is applied to the device. When the negative voltage reaches −1.2 V, the current decreases rapidly and the device switches to the high resistance state (HRS). Subsequently, under application of a positive voltage ranging from 0 to 2.5 V, the device can switch back to the LRS, which corresponds to the set state. We successfully repeated this switching process for 800 cycles through DC voltage sweeps. The endurance of the HRS and the LRS is shown in Fig. 3b. The device shows good reliability at a read voltage of 0.5 V. Figure 3c shows the retention characteristics of this device at room temperature and at 80 °C. After a retention time of 10^5^ s, the HRS and LRS currents are still equal to that of the initial state. The *I-V* characteristics of 30 cycles are presented in Fig. 3d. It is shown that the device maintains a similar on/off ratio and shows good reliability. The observed fluctuations may be caused by random migration of defects during RS cycling, and the fluctuation becomes more prominent when the programming current is ultra-low^18^. The small asymmetry between the positive and negative *I-V* characteristics is related to the variation of the work functions of the top and bottom electrodes, which induces different tunneling barriers in LRS conduction. The work function of the top Al electrode is 4.2 eV, which is smaller than that of the bottom p^+^-Si electrode, at approximately 5.0 eV. Therefore, the positive current may be smaller than the negative current.

It should be noted that the reset current here is 740 nA, which is less than 1 μA. Under such low current conditions, we observe a reset sweep stop at −0.8 V although it actually stops at 0 V. The reason lies in the production of a positive transient current. We found that a positive transient current exist in our device as the applied negative voltage decreases, which is due to the discharging of trap states in a-SiN_x_:H film^19^. The intensity of transient current is less than 1 pA. When the dc current of our devices is high, this effect of transient current is not obvious. But with the magnitude of negative voltage decreasing to less than 0.8 V, the dc current becomes very lower than that of the transient current. And the effect of positive transient current is then evident. The value of opposite current can-not be plotted in the logarithmic coordinates. Thus the data of current in the scale from −0.8 to 0 V can-not be shown in this form. Furthermore the read current of HRS and LRS at 0.5 V are about pA and nA order, respectively. These parameters here are obviously lower than that of other RRAM. Meanwhile the low set voltage (~2.5 V) and reset voltage (~−1.6 V) confirm that an ultra-low power resistive switching is now realized in the Al/a-SiN_1.17_:H/p^+^-Si structure. I-V characteristic of forming process with negative voltage is shown in the inset of Fig. 3d, which is almost identical to the positive curves except a little bit higher voltage. The observed RS behavior in two directions proves that ultra-low power RS originate solely from a-SiN_x_:H film itself.

To assess the uniformity of the a-SiN_x_:H devices, the statistical distributions of the operating voltages and currents are shown in Fig. 3e. The devices show good uniformity with small deviations. We also measured the size dependence characteristics of the devices. It is found the HRS and LRS current decrease with electrode size reduced from 300 μm to 30 μm, as shown in the inset of Fig. 3f. Figure 3f also shows the bipolar RS behavior when the size decreased to 30 μm, and the programming current can be reduced to less than 100 nA with the operating voltage increased to 3.2 V. We see that the reset sweep seems to stop at −1 V, which indicates the effect of transient current. The increased operation voltage may be related to the wet etching process for Al electrode with the smaller diameter of 30 um. Interfacial contact between the Al electrode and SiN_1.17_:H film is easily affected during the wet etching process because the a-SiN_x_:H film thickness is only 8 nm. Overall, the operation power can be reduced by down-scaling of device size.

### Temperature dependence of current in the a-SiN_1.17_:H device

**Figure 4:**
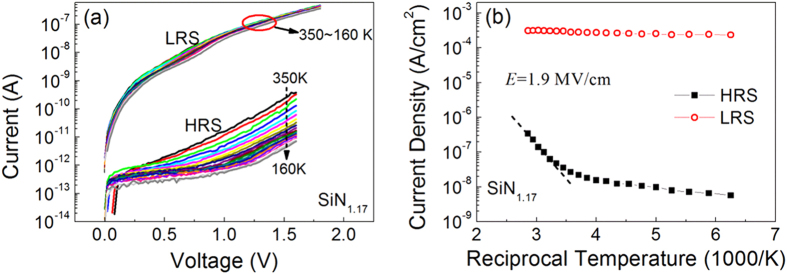
Temperature dependence of a-SiN_x_:H device current. (**a**) *I-V* curves of HRS and LRS at temperatures ranging from 160 K to 350 K. (**b**) Temperature dependence of current density under an electric field of 1.9 MV/cm.

We also measured the temperature dependence of the HRS and LRS currents in the analysis of the conduction process of the SiN_1.17_:H device, as shown in Fig. 4a. The current of the HRS clearly decreases with decreasing temperatures from 350 K to 160 K. This indicates that the conduction of HRS is sensitive to temperature change, i.e., it is a thermal-activated conduction process. The temperature dependence of the current density under an electric field of 1.9 MV/cm is shown in Fig. 4b. Based on the slope of the ln *J* vs 1/*T*, the activation energy of HRS at high temperature is calculated to be 0.4 eV. The temperature dependence of HRS current here is unlike that in usual RRAM devices, where slightly temperature dependence is observed^4^,^20^. In usual RRAM devices with much higher current, the HRS is usually formed due to rupture of conductive filament and the conduction is dominated by tunneling process. Tunneling conduction is independent on temperature, so slightly temperature dependence can be observed in usual RRAM devices. In contrast to the HRS current of ultra-low power device, the LRS current remains the same with decreasing temperature from 350 K to 160 K, as shown in Fig. 4a, which means that LRS is insensitive to temperature changes. This reveals that LRS in the ultra-low power device is dominated by tunneling process instead. It is verified that the characteristics of LRS are not related to the metal filaments, where the LRS resistance generally increases with increasing temperature.

### Atomic configurations of a-SiN_x_:H films with different N/Si ratios

**Figure 5:**
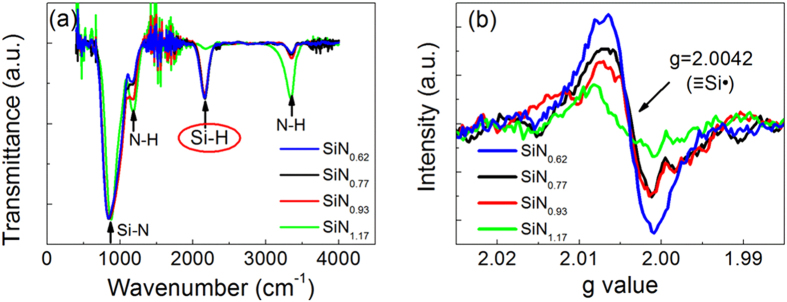
(**a**) FTIR and (**b**) ESR spectra of a-SiN_x_:H devices with different N/Si ratios. The concentration of Si-H and Si dangling bonds decreased with increasing N/Si ratio.

To investigate the relationship between the mechanism and the configurations of a-SiN_x_:H films with various N/Si ratios, we analyzed the corresponding Fourier transform infrared (FTIR) spectroscopy and electron spin resonance (ESR) spectra as shown in Fig. 5. The absorption bands at 841 cm^−1^, 1176 cm^−1^, 2173 cm^−1^ and 3356 cm^−1^ correspond to the Si-N stretching, N-H rocking, Si-H stretching, and N-H stretching modes, respectively^21^. The Si-H and N-H bonds are derived from the hydrogenation of silicon nitride. It is clearly shown that the intensity of the Si-H bonds in films with x = 0.62–0.93 is much higher than that in sample with x = 1.17. This can be ascribed to the increased Si concentrations in films with lower x values. The excess Si atoms combine with H atoms to form more Si-H bonds in the films. In all the a-SiN_x_:H films with various N/Si ratios, we observed a resonance peak with a g value of 2.0042 as shown in Fig. 5b, which is related to the paramagnetic center of the Si dangling bonds 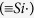
22. It is also found the resonance peak intensity increases with decreasing x value. And the a-SiN_1.17_:H film has the lowest resonance peak. The weak Si dangling bonds signal in SiN_1.17_:H shows that few original Si dangling bonds remain in the film. However, for films with lower N/Si ratios, the intensity of the resonance peak is stronger, indicating that more Si dangling bonds exist in these films. The calculated density of Si dangling bonds changes from 1 × 10^17^ to 5 × 10^17^ cm^−3^ as x varies from 1.17 to 0.62. This implies that the Si dangling bonds can be controlled by tuning N/Si ratio.

### Dehydrogenation effect on initial current

**Figure 6:**
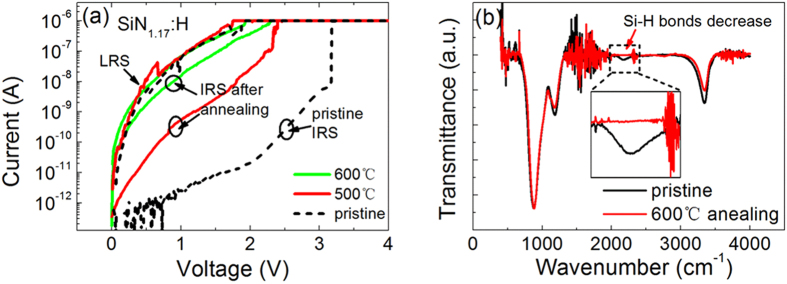
(**a**) Initial current followed by forming process of a-SiN_1.17_:H device before and after dehydrogenation treatment. The initial current increases obviously after dehydrogenation. (**b**) FTIR spectra of the film before and after dehydrogenation.

In order to demonstrate the role of H in our devices, we measured the initial currents of two devices after dehydrogenation treatment, with results as shown in Fig. 6a. The two devices were annealed at 500 °C and 600 °C for 10 min in vacuum. Dehydrogenation generally occurs at 400 °C, so the annealing temperatures and time used here ensures the release of hydrogen. The reduction of H content was confirmed by the FTIR spectrum shown in Fig. 6b. The disappearance of the absorption peak of Si–H stretching vibration mode at 2173 cm^−1^ indicates that a proportion of the hydrogen atoms have effused from the SiN_1.17_:H films. When compared with that of the pristine device, the intensity of initial current for the devices annealed at 500 °C increases sharply and becomes close to that of LRS current. Furthermore, for the device annealed at 600 °C, the intensity of IRS current is nearly as high as that of the LRS current. The increase in the initial current is ascribed to increments in the number of dangling bonds or trap states induced by dehydrogenation^23^. The similar trends for initial current change in the device after dehydrogenation and the device under an applied electric field reveals that H atoms in SiN_1.17_:H films play a key role in the RS process. It is clearly observed that the number of Si-H bonds decreases with increasing N/Si ratio, as verified by the FTIR spectra shown in Fig. 5a, while the number of N-H bonds increases with increasing N/Si ratio. Because the N-H bonds are stronger and more stable than Si-H bonds, the Si dangling bonds are mainly generated from broken Si-H bonds. When more Si atoms exist, then more dangling bonds will be produced. Therefore, a tunable dangling bond path can be obtained by variation of N/Si ratio with the assistance of H atoms.

## Discussion

**Figure 7:**
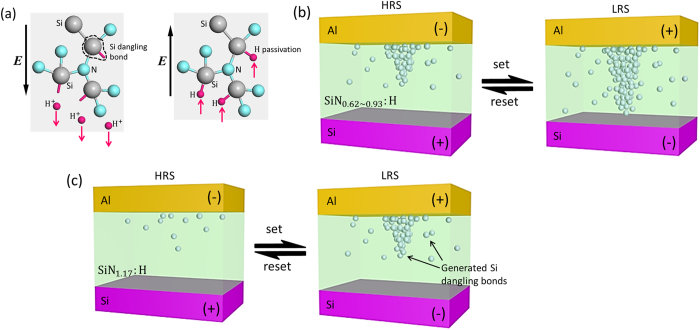
Schematic illustration of RS mechanism in the Al/a-SiN_x_:H/Si device. (**a**) Principle of Si dangling bonds generation and re-passivation process. The applied electric field reduces the activation energy required for thermal bond breakage and finally breaks the weak Si-H bonds. Then with reverse voltage applied, the breakaway H^+^ ions migrate back and re-passivate the Si dangling bonds. (**b**) Si dangling bond conduction path in device with x = 0.62–0.93. The Si dangling bonds act like traps and carriers can flow through these traps by trap to trap tunneling. (**c**) For device with x = 1.17, fewer Si dangling bonds are generated in LRS and the conduction path dissolves completely in HRS.

As revealed by the FTIR and ESR that there are many Si-H bonds and a few Si dangling bonds in the pristine a-SiN_x_:H films. Si-H bonds can be broken during the forming or set process due to the field-enhanced thermal breakage^24^,^25^, because the Si-H bond energy (3.0 eV) is much lower than that of Si-N (3.7 eV)^24^. The H^+^ ions from broken Si-H bonds migrate toward the cathode under the positive electric field, producing many new Si dangling bonds in the film. A schematic illustration of this process is shown in Fig. 7a. The Si dangling bonds are neutral traps near the middle of the bandgap and can form a conductive path when the number of dangling bonds achieves a certain level. The carriers can flow through the Si dangling bond conduction path by tunneling process. In our previous work^26^, the similar process is demonstrated in SiO_x_ film. The Si-O bonds could be broken to form Si dangling bonds at high electric field through standard Boltzmann process. In the a-SiN_x_:H film, the operation voltage is much lower than that of SiO_x_ film because the Si-H bond energy is weaker than that of Si-O bonds. As the negative voltage was applied, the H^+^ ions can migrate back and re-passivate the Si dangling bonds with the aid of thermal effect, leading to the decrease of Si dangling bonds. Si dangling bond pathway thus ruptures and the device returns to the HRS.

Based on the above analysis, we will explain how the programming current can be controlled by tuning Si dangling bond conduction paths. As illustrated in Fig. 5 the pristine devices with higher N/Si ratios initially contain fewer Si-H bonds, which results in far fewer Si dangling bonds being generated during the forming process. The conduction path containing fewer Si dangling bonds will produce lower currents for LRS. Figure 7b,c shows the schematic diagram of Si dangling bond conduction path in the devices with different N/Si ratio, respectively. As for the sample with x = 0.62–0.93 shown in Fig. 7b, the Si dangling bonds are partially re-passivated and the conduction path ruptures in HRS. When N/Si ratio increases to 1.17, the conduction path can not pass through the whole film because of the low Si dangling bonds density. It is observed that the LRS current in a-SiN_1.17_:H device is close to the HRS current in SiN_0.93_:H device as shown in Fig. 2b. Furthermore the conduction path nearly dissolves in HRS as presented in Fig. 7c, because the HRS current in a-SiN_0.93_:H device is close to IRS current. Consequently, the Si dangling bond conduction path can be tuned by controlling N/Si ratio of the a-SiN_x_:H films.

**Figure 8:**
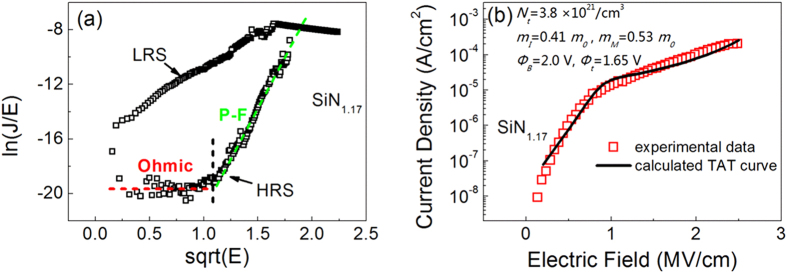
(**a**) Plot of *I-V* curve of a-SiN_1.17_:H device with ln (*J*/*E*) versus 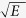 according to the P–F model. (**b**) Comparison between the experimental and calculated *I-V* curve for the LRS in a-SiN_1.17_:H device based on the TAT model. The parameters used in the calculation are shown at the top of this figure.

**Figure 9:**
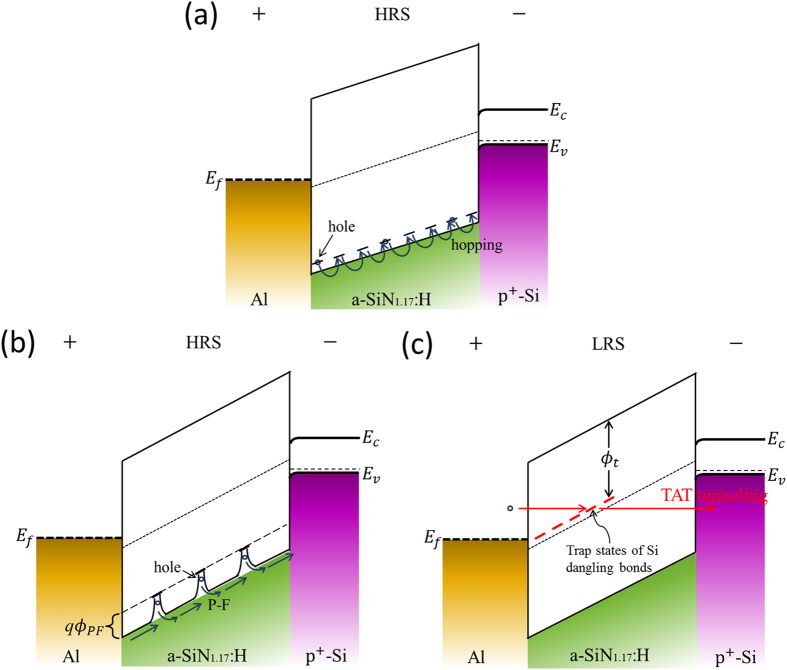
Energy band diagram of conduction in (a) HRS under a low electric field, (b) HRS under a high electric field and (c) LRS for the ultra-low power a-SiN_1.17_:H device. In HRS, the conduction is dominated by hopping and P–F process from trap at approximately 0.9 eV above the valence band. In LRS, the conduction is dominated by TAT conduction with Si dangling bonds as intermediate trap states.

According to the analysis of atomic configuration of a-SiN_x_:H film with different N/Si ratio, we perform theoretical calculations to investigate the conduction mechanism of the proposed ultra-low power device. As reported in the literatures about conduction mechanism of silicon nitrides^27^,^28^,^29^, the main carriers in a-SiN_x_:H films are holes and the current conduction mechanism is dominated by hopping between localized states under low electric fields and Poole–Frenkel (P–F) process under higher electric fields. The current density versus electric field relationship in the P–F process is expressed as^28^


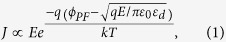


where *E* is electric field, *J* is the current density, *q* is the electronic charge, 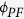 is the barrier height of trap, *ε*_0_ is the permittivity of free space, *ε*_*d*_ is the dynamic dielectric constant, *k* is the Boltzmann constant, and *T* is the temperature. We agree that the IRS of all our devices obey this conduction model. For the HRS of SiN_1.17_:H device, we plot the set curve with ln (*J*/*E*) against 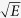 as shown in Fig. 8a. When the electric field *E* < 1.25 MV/cm, ln (*J*/*E*) is constant and independent of the electric field, which indicates ohmic conduction in this region. This corresponds to the hopping process under low electric fields. Then, when E > 1.25 MV/cm, the straight line of the plots implies the dominant role of P–F process. According to equation (1), the value of dynamic dielectric constant 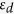 can be estimated from the slope of ln (*J*/*E*) –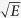 plot. Here the value of *ε*_*d*_ is 4.2, which is between the dielectric constant in the optical range, 4 and the static dielectric constant 7. As pointed out by S.M. Sze^28^, the value of *ε*_*d*_ is of paramount importance, which confirms that the HRS is dominated by P–F process. This conduction model is consistent with the current temperature dependence shown in Fig. 4, because P–F effect is a thermal activated conduction process. By entering the activation energy 0.4 eV into equation (1), the potential well *q*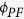 of the trap in P–F effect is determined to be 0.9 eV. The energy band diagram of the conduction process in HRS is presented in Fig. 9a,b.

On the other hand, the LRS plots are also found to be close to a straight line, but the calculated *ε*_*d*_ is about 22.6, which is far beyond the scope above. Considering that LRS is insensitive to temperature change, we conjecture that the conduction of LRS should be dominated by tunneling mechanism rather than P–F process. We firstly exclude the probability of Fowler–Nordheim (F–N) tunneling model for LRS conduction in a-SiN_1.17_:H device. The F–N tunneling is due to current tunneling through the triangular barrier with a high electric field (>6 MV/cm). However, the LRS current here were measured under electric field less than 3 MV/cm. So the electric field is too low to generate F–N tunneling current. Alternatively, if the insulation film is very thin (<4 nm), the electrons can flow through the film by direct tunneling under low electric field. However the SiN_1.17_:H film is 8 nm thick, and thus the possibility of direct tunneling can also be eliminated. Recently the trap-assisted tunneling (TAT) model has been used to explain the stress-induced leakage current in nitrided oxide films^30^,^31^,^32^,^33^, which includes two-step tunneling process via traps in the film. The carriers first tunnel into the trap state from one electrode, and then tunnel into the other electrode. The tunneling current can be generated under low electric field even in much thicker film. In the a-SiN_1.17_:H device, the new Si dangling bonds that were created during forming or set process provides the intermediate states for TAT conduction and generate a considerable tunneling current. Therefore the TAT conduction is the most reasonable conduction mechanism for LRS in a-SiN_1.17_:H films. The conductive path is composed of Si dangling bonds. The energy band diagram of this conduction process is shown in Fig. 9c.

In order to further verify whether or not the Si dangling bond conduction paths obey the TAT conduction model, we give a calculation to fit this model and clarify the trap energy level in our device. We use a generalized thermionic TAT model to describe the tunneling process in LRS^33^. Under low electric fields in this model, the tunneling occurs through a trapezoidal barrier. While under high fields tunneling through the triangular barrier becomes more significant. In our devices the measured voltage of LRS is less than 2 V, so we only have to take the trapezoidal barrier tunneling into account. As shown in Fig. 9c, the holes tunnel from Al top electrode to the trap state of Si dangling bonds in the film, and then tunnel to the valence band of p^+^-Si. The equation for this current is given as^33^


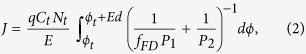


where *N*_*t*_ is the trap concentration, *ϕ*_*t*_ is the trap energy level, *d* is the film thickness, and *f*_*FD*_ is the Fermi-Dirac function, which is described by


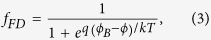


*ϕ*_*B*_ is the barrier height between electrode and film. *P*_1_ and *P*_2_ are the tunneling probabilities for the two-step process, where


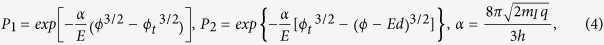


and *C*_*t*_ is the trap energy dependent rate constant that is given by


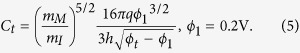


We calculated the *I-V* curve using equation (2) for comparison with experimental LRS data as shown in Fig. 8b. The parameter values used in calculation are shown inside this figure. It is notable that the *ϕ*_*t*_ value is 1.65 V. This value is close to the energy level of Si dangling bonds in the silicon nitride film^29^,^34^, which theoretically confirms that the generated trap states originate from Si dangling bonds. Therefore LRS in the ultra-low power device is dominated by the TAT conduction with Si dangling bonds as the intermediate trap states. Here the value of trap concentration Nt is 3.8 × 10^21^ cm^−3^ because sufficient trap centers are needed to produce a considerable TAT current. As reported in ref. 33, a higher trap density of 6 × 10^22^ cm^−3^ was used to calculate the TAT current. Although the trap density is high in low power device, the current is lower than that of SiN_0.62~0.93_:H devices because the conduction path is not continuous and it is dominated by two-step tunneling process. As for the SiN_0.62~0.93_:H devices, the trap density should be much higher and the current is dominated by continuous conduction.

In conclusion, we demonstrate that ultra-low power Si-based bipolar resistive switching memory devices can be realized in Al/a-SiN_x_:H/p^+^-Si structure by tuning Si dangling bond conduction paths. The relationship between Si dangling bonds and the N/Si ratio x for a-SiN_x_:H films reveals that programming current can be reduced to less than 1 μA with operating voltage of less than 2.5 V by increasing the value of x. We found that the field-enhanced thermal breakage of weak Si-H bonds and migration of the H^+^ ions result in the generation and re-passivation of Si dangling bonds in a-SiN_x_:H films. Consequently tunable dangling bond paths can be obtained by varying the N/Si ratio. Our theoretical calculation of *I-V* curves for the LRS indicates that the energy level of LRS trap states is in close agreement with the value of Si dangling bond, which confirms that Si dangling bond conduction paths dominate the TAT model. While, for HRS the conduction is dominated by either hopping or P–F process. Our introduction of hydrogen to the resistive switching a-SiN_x_ film provides a new way to control the Si dangling bond conduction path, and this opens up a potential research field for ultra-low power Si-based RRAM.

## Methods

The a-SiN_x_:H films in this paper were deposited on p^+^-Si substrates in a plasma-enhanced chemical vapour deposition (PECVD) system operating at 250 °C, with SiH_4_ and NH_3_ as the reaction gases. To form the top electrode, 300-nm-thick aluminum with a diameter of 300 μm were deposited on the top of the films using thermal evaporation method. The smaller top electrode with diameter of 30 and 100 um was fabricated by lithographic method combined with wet etching. The rear of the substrate was also coated with a thin Al layer to reduce the contact resistance. The entire fabrication process is very simple and does not require any high-temperature treatment. To reduce the programming current, samples with four different Si/N ratios were prepared by varying the flow rate ratio of SiH_4_ and NH_3_. The N/Si atomic concentration ratios of the samples are determined through XPS measurement using the PHI 5000 VersaProbe. The microstructure of the sample was revealed by HRTEM using a Tecnai G^2^ F20 electron microscope operating at 200 kV. The ESR spectrum was measured in the Bruker EMX-10/12 system and FTIR spectrum was measured in the NEXUS870 system. The dehydrogenation process was carried out by annealing treatment at 500 °C and 600 °C for 10 min in vacuum, respectively. The electrical characteristics of the samples were measured using the Agilent B1500A semiconductor analyzer in atmosphere, and the temperature dependence of the *I-V* characteristics was examined in the Lake Shore CRX-4K system under a vacuum of 5 × 10^−5^ Torr.

## Additional Information

**How to cite this article**: Jiang, X. *et al.* a-SiNx:H-based ultra-low power resistive random access memory with tunable Si dangling bond conduction paths. *Sci. Rep.* **5**, 15762; doi: 10.1038/srep15762 (2015).
